# Multivariate stabilizing sexual selection and the evolution of male and female genital morphology in the red flour beetle*

**DOI:** 10.1111/evo.13912

**Published:** 2020-01-23

**Authors:** Clarissa House, Philip Tunstall, James Rapkin, Mathilda J. Bale, Matthew Gage, Enrique del Castillo, John Hunt

**Affiliations:** ^1^ School of Science and Health and Hawkesbury Institute for the Environment Western Sydney University Richmond NSW 2753 Australia; ^2^ Centre for Ecology and Conservation, College of Life and Environmental Sciences University of Exeter Cornwall TR10 9EZ United Kingdom; ^3^ School of Biological Sciences University of East Anglia Norwich NR4 7TJ United Kingdom; ^4^ Department of Industrial Engineering and Department of Statistics Pennsylvania State University State College Pennsylvania 16802

**Keywords:** Fitness peak, genitals, lock‐and‐key hypothesis, selection analysis

## Abstract

Male genitals are highly divergent in animals with internal fertilization. Most studies attempting to explain this diversity have focused on testing the major hypotheses of genital evolution (the lock‐and‐key, pleiotropy, and sexual selection hypotheses), and quantifying the form of selection targeting male genitals has played an important role in this endeavor. However, we currently know far less about selection targeting female genitals or how male and female genitals interact during mating. Here, we use formal selection analysis to show that genital size and shape is subject to strong multivariate stabilizing sexual selection in both sexes of the red flour beetle, *Tribolium castaneum*. Moreover, we show significant sexual selection on the covariance between the sexes for specific aspects of genital shape suggesting that male and female genitalia also interact to determine the successful transfer of a spermatophore during mating. Our work therefore highlights the important role that both male and female genital morphologies play in determining mating success and that these effects can occur independently, as well as through their interaction. Moreover, it cautions against the overly simplistic view that the sexual selection targeting genital morphology will always be directional in form and restricted primarily to males.

Male genitals in animals with internal fertilization are widely regarded as being among the most divergent and variable of all morphological structures, to the extent that genital morphology is often used to distinguish between closely related species that are otherwise indistinguishable (reviewed in Hosken and Stockley 2004; Simmons 2014). Not surprisingly, the proximate mechanism(s) responsible for this variation has puzzled evolutionary biologists for several decades and has been the topic of investigation in almost all animal taxa (e.g., insects: House and Simmons 2003; House et al. 2013; spiders: Foellmer 2008; Kuntner et al. 2016; reptiles: King et al. 2009; fish: Langerhans et al. 2005; Booksmythe et al. 2016; birds: Brennan et al. 2010, 2017; mammals: Stockley 2002; Ramm 2007). More recently, there have been a growing number of studies showing that female genitals may often be as variable as male genitals and may also evolve as rapidly (e.g., Simmons 2014; Ah‐King et al. 2014). Despite this, there is a strong under‐representation of studies on female genitalia (Ah‐King et al. 2014), as well as a general lack of studies examining how complex interactions between the sexes can shape genital coevolution (Ah‐King et al. 2014; Brennan and Prum 2015). A more detailed understanding of genital evolution therefore requires a greater focus on female genitals and how they interact with male genitals during mating (Ah‐King et al. 2014; Brennan and Prum 2015).

Historical explanations for the evolution of genitals have largely focused on three main hypotheses: the lock‐and‐key, the pleiotropy, and the sexual selection hypotheses (Hosken and Stockley 2004). Originally, the lock‐and‐key hypothesis proposes that genital divergence has evolved to prevent hybridization by ensuring that only males of the correct species were able to provide the right “key” for the female “lock” (Dufour 1844). More recently, however, Arnqvist (1997) proposed that a similar process could also operate within a species, with the poor alignment of male and female genitals reducing male mating success. The pleiotropy hypothesis proposes that the divergence in genital morphology is due to the pleiotropic effects of selection on other nongenital traits (Mayr 1963). Thus, genital divergence is considered a neutral process with genitals only evolving because they are genetically correlated with other nongenital traits that are the target of selection (Mayr 1963). Finally, the sexual selection hypothesis proposes that a number of different processes, most notably cryptic female choice for males with genitals that are better able to stimulate them during mating or through sperm competition and/or sexual conflict, all have the potential to drive genital divergence (Hosken and Stockley 2004). Although there is a degree of empirical support for both the lock‐and‐key (e.g., Wojcieszek and Simmons 2012, 2013; Anderson and Langerhans 2015) and pleiotropy (e.g., Arnqvist and Thornhill 1998; House and Simmons 2005) hypotheses, most support is currently for the sexual selection hypothesis (e.g. Hosken and Stockley 2004; Ah‐King et al. 2014; Simmons 2014). This is not altogether surprising given the much larger number of studies testing this hypothesis (Ah‐King et al. 2014) and highlights the need for more empirical studies focused on the lock‐and‐key and pleiotropy hypotheses.

One important criterion that has been used to discriminate between these alternate hypotheses is the form of selection targeting male genitals (Arnqvist 1997; Hosken and Stockley 2004). According to the original lock‐and‐key hypothesis, it is predicted that a pattern of stabilizing natural selection prevents hybridization by ensuring that male genitals are species specific and provide the correct “key” to fit the female “lock” (Dufour 1844). Moreover, within a species, the reduction in mating success that occurs when male genitals are poorly aligned with the average genital structure of females in the population is predicted to result in a pattern of stabilizing sexual selection targeting male genitals (Arnqvist 1997). Thus, although both scenarios predict that male genital morphology will be under stabilizing selection, the mode of selection is fundamentally different (i.e., natural vs. sexual selection). According to the sexual selection hypothesis, variation in male genital morphology is related to fertilization success, with males having extreme genitals being the most successful due to their stimulatory, competitive, and/or coercive ability (Arnqvist 1997). Sexual selection is therefore predicted to impose strong linear (or directional) selection on male genitals (Arnqvist 1997; Hosken and Stockley 2004). In contrast, according to the pleiotropy hypothesis, male genital morphology does not correlate with fitness and therefore should not experience any direct selection (Arnqvist 1997). However, if phenotypically correlated with other traits under selection, male genitals can experience indirect selection and this can take any form (Arnqvist 1997).

The ability to empirically quantify the strength and form of selection acting on male genitals has been greatly enhanced by the use of multivariate selection analysis (Lande and Arnold 1983) and insects have played a key role in this endeavor. The majority of studies on insects have documented linear selection on male genitals (e.g., damselflies: Cordoba‐Aguilar 1999, 2002, 2009; water strider: Arnqvist and Danielsson 1999; Danielsson and Askenmo 1999; Bertin and Fairbairn 2005; praying mantis: Holwell et al. 2010; oriental beetle: Wenninger and Averill 2006; earwig: van Lieshout 2011; van Lieshout and Elgar 2011), although stabilizing selection has been shown to target some aspects of male genital morphology as well (seed bug: Tadler 1999; Dougherty and Shuker 2016; dung beetle: Simmons et al. 2009; millipede: Wojcieszek and Simmons 2012; broad horned beetle: House et al. 2016; water strider: Bertin and Fairbairn 2005). Although this finding appears to support the sexual selection hypothesis, it is important to note that it is easier to statistically detect linear than stabilizing selection (Lande and Arnold 1983), and that there are also many empirical examples showing that sexual selection is not always linear in form (e.g., Blows et al. 2003; Chenoweth and Blows 2005; Brooks et al. 2005; Bentsen et al. 2006; Gerhardt and Brooks 2009; Steiger et al. 2013). Unfortunately, we currently lack similar formal estimates of selection for female genital morphology. In general, more comprehensive studies are needed (ideally exceeding several hundred individuals; Hersch and Phillips 2004) to characterize the pattern of linear and nonlinear selection acting on genital morphology, especially for females.

The red flour beetle, *Tribolium castaneum* (Coleoptera: Tenebrionidae), is a model species in the study of sexual selection (reviewed in Fedina and Lewis 2008). This species is highly polygamous and will mate every few minutes, yet as high as 55% of mating attempts do not produce viable offspring (Lewis and Iannini 1995; Bloch Qazi et al. 1996; Pai et al. 2005; Fedina and Lewis 2008). Although pericopulatory processes are likely to explain this outcome (Tyler and Tregenza 2012), the role that male and female genital morphology (or their interaction) play in the successful transfer of a spermatophore remains unknown. Here, we use multivariate selection analysis (Lande and Arnold 1983) to characterize the strength and form of direct linear and nonlinear sexual selection acting independently on male and female genital size and shape in the red flour beetle, *T. castaneum*. Having shown that the fitness surfaces for both sexes contain a peak, we then formally test whether this represents stabilizing selection. It has been argued that a fitness surface containing a peak should only be referred to as stabilizing selection if the peak resides within the phenotypic space sampled (Mitchell‐Olds and Shaw 1987). To test this, we estimated the location of the global maxima and the 95% confidence region (CR) for each sex and mapped these alongside the fitness surfaces displaying the individual measures of genital size and shape. Finally, we estimated the sign and strength of correlational selection that targets the covariance between male and female genital size and shape. The standardized selection gradients from this analysis therefore measure the importance of the interaction between male and female genital morphology for the successful transfer of a spermatophore during mating.

## Materials and Methods

### STOCK POPULATIONS AND REARING PROTOCOL

A total of six stock populations (∼200 beetles per population) of the widely used Georgia 1 (GA1) “wild‐type” strain of *T. castaneum* were originally derived from the Beeman Lab (US Grain Marketing Production Research Centre). These populations were cultured in ad libitum standard medium (95% white flour and 5% bakers’ yeast) and maintained at 30°C, 60% humidity and on a 16:8 h light:dark cycle for three generations prior to the start of our experiment. Each generation, adult beetles were mixed at random between populations to ensure gene flow and the maintenance of genetic variation.

### EXPERIMENTAL PROCEDURE

Male and female beetles used in this experiment were taken at random from the stock populations. To ensure virginity, pupae were collected from the stock populations over a two‐week period. A set of nested sieves were used to separate the pupae from the adults and medium. The pupae were then removed from the sieve with soft grip tweezers and their sex was determined under a microscope. Each pupa was then placed into an individual cell of a unisex, square plastic transparent box (10 cm^2^; 25 cells per box, 2 cm^2^ per cell), with each cell half‐filled with medium. The boxes were checked daily and the eclosion date for emerging adults was recorded to ensure that only virgin adults aged 7–21 days were used in the mating trials (Attia and Tregenza 2004).

### MATING TRIALS

Mating trials were conducted at 22 ± 1°C in a mating arena that consisted of 2 × 2 cm cells in a 25 cell box that was lined with paper to provide traction (Tyler and Tregenza 2012). In every trial, a female was first placed into one of the mating arena cells followed by a male. The time of male introduction and the start and end of mating was recorded. Males typically make multiple mounting attempts; however, we define mating as a mounting that lasted longer than 30 s, as shorter mating attempts are unlikely to result in the transfer of a spermatophore (Tyler and Tregenza 2012). Following a mating longer than 30 s, the male was removed and frozen (*n* = 535). To verify whether a mating attempt had been successful or unsuccessful, each female was placed in a 60‐mL breeding pot (sized 67 mm × 34 mm) that contained 30 mL of standard media to oviposit under the standard incubation conditions. After seven days, each female was removed and frozen (*n* = 535). Forty days later, each pot was checked for the presence or absence of offspring (now newly eclosed beetles) to verify whether mating was successful or failed. Mating pairs were classified as successfully mated if mating resulted in offspring and received a fitness score of one (*n* = 216). Mating pairs were classified as unsuccessfully mated if no offspring were produced and received a fitness score of zero (*n* = 282).

### DISSECTIONS AND GEOMETRIC MORPHOMETRICS

The male genitalia were removed from the abdomen and mounted on a microscope slide in a drop of Hoyer's solution. The female genitalia were squeezed out of the body by gently pressing the abdomen and mounted in a drop of Hoyer's solution, while still attached to the body. The genitalia are delicate and prone to damage during dissection. As we are interested in estimating the selection independently targeting male and female genitals, as well as the covariance between these structures during mating, we required intact genitals from both individuals in the interacting mating pair. When damage to the male or female genitals occurred, we removed the mating pair from the dataset leaving a final sample size of 498. All genitalia were placed in a consistent, longitudinal orientation and digital images were taken using a Leica DFC295 digital microscope camera that was mounted on a dissecting Leica M125 microscope (Figure S1 and S2). Due to the complexity of the male and female genitalia, geometric morphometric (GM) analysis was used to quantify the variation in the size and shape of the outline of the male aedeagus and female vagina and supporting structures. A description of the programs used to digitize the male and female genitalia and analyze the GM data is described in Figure S1 and S2. Although our shape analysis for males and females returned a total of 19 and 36 RW scores, respectively, only the first four were used as they each accounted for over 75% of the shape variation (Gutierrez et al. 2011). Our measurements of genital morphology were highly repeatable in both sexes (Table S1).

### STATISTICAL ANALYSIS

#### Characterizing linear and nonlinear sexual selection on male and female genital size and shape

We used standard multivariate selection analysis (Lande and Arnold 1983) to evaluate the strength and form of linear and nonlinear selection acting on male and female genital size and shape. An absolute fitness score was assigned to each male and female in our experiment, with one being assigned to the male and female in a pair that successfully obtained a mating and zero being assigned to the male and female in unsuccessful pairs. Following Lande and Arnold (1983), this absolute fitness score was transformed to relative fitness by dividing by the mean absolute fitness of the population.

To estimate the standardized linear selection gradients (***β***), a first‐order linear multiple regression model was fitted using centroid size (CS) and the first four RW scores describing the variation in male and female genital shape as the predictor variables, and relative fitness as the response variable (Lande and Arnold 1983). We then used a second‐order quadratic multiple regression model that included all linear, quadratic, and cross‐product terms to estimate the matrix of nonlinear selection gradients (**γ**) that describes the curvature of the fitness surface. Quadratic regression coefficients are known to be underestimated by a factor of 0.5 using standard multiple regression analysis, so we doubled the quadratic selection gradients derived from this model (Stinchcombe et al. 2008). In addition to these conventional models for each sex, we also fit a second multiple regression model to account for the interaction between male and female genitals during mating. In this model, we first estimated ***β*** using a first‐order linear multiple regression model that included CS and the four RW scores for each sex as the predictor variables, and relative fitness as the response variable. We then estimated **γ** using a second‐order quadratic multiple regression model that included all linear, quadratic, and cross‐product terms for each sex, as well as cross‐product terms between each sex, as the predictor variables and relative fitness as the response variable. It was not our intention to interpret the selection gradients from this analysis but simply to demonstrate that including the genital morphology of both sexes and their interaction in the same model did not alter the sign or strength of the resulting selection gradients compared to the conventional models.

As relative fitness does not conform to a normal distribution, we used a resampling procedure to assess the significance of our standardized selection gradients (Mitchell‐Olds and Shaw 1987). We randomly shuffled relative fitness scores across male and female pairs in our dataset to obtain a null distribution for each selection gradient where there is no relationship between our measures of genital size and shape and relative fitness. We used a Monte Carlo simulation to determine the proportion (*p*) of times (out of 10,000 iterations) that each gradient pseudo‐estimate was equal to or less than the original estimated gradient, and this was used to calculate a two‐tailed probability value (as 2*p* if *P* < 0.5 or as 2(1 – *p*) if *P* > 0.5) for each selection gradient in the model (Manly 1997). We conducted separate randomization tests for the linear multiple regression model and the full quadratic model (including linear, quadratic, and correlational terms) following the procedure outlined above.

As the strength of nonlinear selection gradients can be underestimated by interpreting the size and significance of individual **γ** coefficients (Blows and Brooks 2003), we explored the extent of nonlinear selection acting on male and female genital size and shape by conducting a canonical analysis of the **γ** matrix to locate major eigenvectors of the fitness surface in each sex (Phillips and Arnold 1989). For each sex, we used the permutation procedure outlined in Reynolds et al. (2010) to determine the strength and significance of nonlinear selection operating along the eigenvectors of **γ**. This procedure, however, does not estimate the strength of linear selection operating along the eigenvectors of **γ** and we therefore used the “double regression” method of Bisgaard and Ankenman (1996) to estimate this form of selection acting along each eigenvector. The strength of linear selection along each eigenvector (**m*_i_***) is given by theta (***θ_i_***), whereas the strength of nonlinear selection is given by their eigenvalue (**λ*_i_***).

We used thin‐plate splines (Green and Silverman 1994) to visualize the major eigenvectors of the fitness surface extracted from the canonical rotation of the **γ** for males and females. We used the “*Tps*” function in the FIELDS package of R (version 2.13.0, www.r-project.org) to fit the thin‐plate splines, and visualized splines as a contour map using the value of smoothing parameter (λ) that minimized the generalized cross‐validation score (Green and Silverman 1994).

#### Estimating the location of the global maxima on the fitness surface and its 95% CR

According to Lande and Arnold (1983), a phenotypic trait subject to stabilizing selection is characterized by a negative standardized quadratic selection gradient (i.e., **γ** < 0). The problem with this definition is that a negative value of **γ** does not guarantee that a peak in fitness will exist within the range of existing phenotypes sampled (Mitchell‐Olds and Shaw 1987). This scenario could arise, for example, if there is a monotonic increase in fitness across the range of phenotypes examined, meaning the location of the fitness peak must be based purely on extrapolation (Mitchell‐Olds and Shaw 1987). Mitchell‐Olds and Shaw (1987) therefore proposed that stabilizing selection should be limited to cases where extreme phenotypes have lower fitness and maximum fitness occurs at some intermediate point in the phenotypic distribution. Although Mitchell‐Olds and Shaw (1987) provide a number of statistical tests to support this definition of stabilizing selection, a simpler approach is to visually compare the location of the fitness peak to the distribution of the phenotypic data. To this end, we estimated the location of the global maxima (fitness peak) and the 95% CR for the fitness surface of each sex and mapped these alongside the same fitness surfaces displaying the individual measures of genital size and shape.

Existing methods for finding the CR associated with the location of the maxima of a regression function rely on the assumption that the data are normally distributed (Peterson et al. 2002), which is clearly not the case for our measure of fitness. Here, we use nonparametric bootstrapping method that we have previously developed (del Castillo et al. 2016) that is not based on any distributional assumptions and can find CRs on the location of the maxima of either quadratic polynomial models or of more flexible thin‐plate spline models. This approach is provided by the “OptRegionTps” function in the OPTIMAREGION package of R (del Castillo et al. 2016).

In brief, a quadratic polynomial model was fit to the data using ordinary least squares regression implemented in the “lm” function of R, yielding a fitted response surface $\hat{y}\left(x\right)$ and residuals $r_{i}=\;y\left(x\right)-\;\hat{y}\left(x\right)$. We then applied bootstrapping to the residuals to create bootstrapped realizations $y^{*}\left(x\right)\;$= $\hat{y}\left(x\right)$ + $r^{*}$ for each data point in our dataset(x). For each simulated set of $y^{*}\left(x\right)$, we fit a quadratic polynomial and found parameter estimates $\gamma^{*}$. Following Yeh and Singh (1997), we repeated this procedure 1000 times and computed Tukey's data depth for each generated $\gamma^{*}$ vector, keeping the 100(1−α)% deepest (where in our case *a* = 0.95). This provides an approximate nonparametric bootstrap 95% CR for the quadratic polynomial coefficients (γ). The responses $y^{*}\left(x\right)$ that corresponded to the parameter vectors $\gamma^{*}$ lying inside of their CR were then maximized numerically using the NLOPTR package of R (Johnson 2014; Ypma 2014) with respect to the regressors $\left(x_{1},x_{2}\ldots xn\right)$ yielding the bootstrapped response global maxima $\left(x^{*}\right)$. The nonparametric bootstrapped CR for the location of the global maximum of the fitness function is computed as the convex hull of all the bootstrapped maxima $\left(x^{*}\right)$ that were found. We use the centroid (average) of all the maxima found as our point estimate of the global maxima of the fitness surface.

#### Characterizing sexual selection on the interaction between male and female genital morphology

Because we measured the genital size and shape of both males and females in each interacting pair, as well as the outcome of this interaction, we were able to estimate the sign and strength of the correlational selection operating on the covariance between these traits across the sexes. We estimated these between‐sex correlational gradients by fitting a multiple regression model that included the standardized linear, quadratic and cross‐product terms for each sex, and the standardized cross‐product terms between the sexes as the predictor variables and relative fitness as the response variable. As these gradients essentially measure how genital size and shape interact between the sexes to determine fitness, we refer to the resulting between‐sex covariance matrix from this analysis as the interaction matrix and consider the terms in this matrix as being analogous to conventional Lande and Arnold (1983) selection gradients for subsequent interpretation. We used the resampling and thin‐plate spline procedures outlined above to test the statistical significance and to visualize the standardized correlational selection gradients, respectively.

### RESULTS

**Figure 1:**
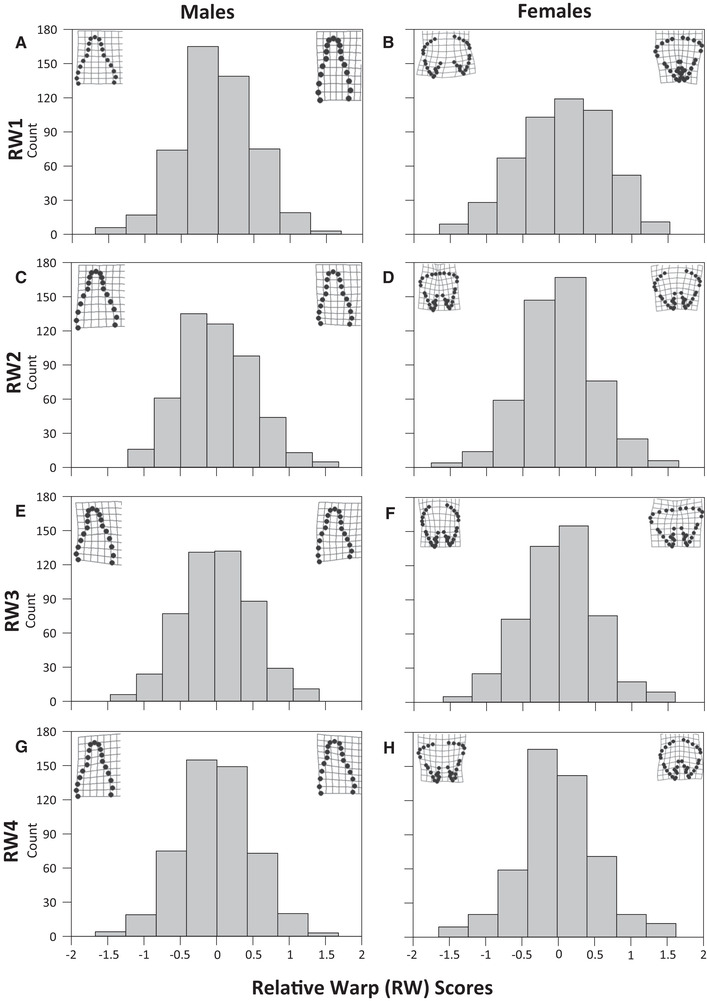
Frequency distribution of the four relative warp (RW) scores characterizing the variation in male (A, C, E, and G) and female (B, D, F, and H) genital shape. For each RW, we provide thin‐plate spline visualizations (inset) that characterize a positive and negative score.

GM analyses yielded a measure of CS and four RW scores for each sex that together explained 78.40% and 85.38% of the total variation in male and female genital shape, respectively. In males, RW1 explained 39.25% of the total variance in genital shape with negative values corresponding to a short, wide aedeagus and positive values to a long, narrow aedeagus (Fig. 1A). RW2 explained a further 15.04% of this total variance with negative values of RW2 corresponding to an anteriorly shortened tip of the aedeagus and positive values to an anteriorly lengthened tip (Fig. 1C). RW3 explained 12.79% of the total variance in male genital shape with negative values corresponding to an anticlockwise twist of the anterior tip of the aedeagus and positive values to a clockwise twist of the anterior tip of the aedeagus (Fig. 1E). RW4 explained the remaining 11.32% of the total variance in male genital shape with negative values corresponding to a compression of the left‐side, posterior of the aedeagus and positive values to a similar compression but on the right‐side of the aedeagus (Fig. 1G).

In females, RW1 explained 59.26% of the total variance in genital shape with negative values corresponding to a wide, short vaginal aperture and positive values to a narrow, elongated vaginal aperture (Fig. 1B). RW2 explained a further 14.26% of the total variation in female genital shape with negative values corresponding to narrower, longer supportive structures of the vagina and positive values to broader, curved supportive structures (Fig. 1D). RW3 explained 7.82% of the total variance in female genital shape with negative values corresponding to an extreme posterior elongation of the supportive structures of the vagina and positive values to an extreme posterior broadening of these supportive structures (Fig. 1F). RW4 explained the remaining 4.04% of the total variance in female genital shape with negative values corresponding to a shorter, wider vaginal aperture and posterior broadening of the supportive structures and positive values to a narrower, tapering vaginal aperture and posterior elongation of the supportive structures (Fig. 1H).

Standardized linear, quadratic, and correlational selection gradients for genital size and shape in males and females are presented in Table 1, panels A and B, respectively. In males, there was significant linear selection favoring increased values of RW1 (long, narrow aedeagus), RW3 (clockwise twist of the anterior tip of the aedeagus), and RW4 (posterior compression on the right‐side of the aedeagus) (Table 1, panel A). There was also significant negative quadratic selection on CS and RW2 (length of the tip of the aedeagus) (Table 1, panel A). In females, there was significant linear selection favoring reduced values of RW3 (extreme posterior elongation of the supportive structures of the vagina) and RW4 (shorter, wider vaginal aperture and posterior broadening of the supportive structures) (Table 1, panel B). There was also significant negative quadratic selection on CS, RW1 (length and width of the vaginal aperture), and RW4 and negative correlational selection on CS and RW1. Importantly, a full model that included the genital morphology of both sexes and their interaction produced quantitatively similar gradients (Table S2): that is, there were strong positive correlations among the linear (*r* = 0.99, *n* = 10, *P* = 0.0001), quadratic (*r* = 0.99, *n* = 10, *P* = 0.0001), and correlational (correlational: *r* = 0.92, *n* = 20, *P* = 0.0001) selection gradients from our conventional models and this full model.

**Table 1:** The vector of standardized linear selection gradients (***β***) and the matrix of standardized quadratic and correlational selection gradients (**γ**) for successful mating in male and female *Tribolium castaneum*. We provide *r* ^2^ estimates for the linear model used to estimate ***β*** and also for the second‐order quadratic model (that includes all linear, quadratic, and cross‐product terms) to estimate **γ** in each sex

		γ				
	*β*	CS	RW1	RW2	RW3	RW4
A. Male	*r* ^2^ = 0.030			*r* ^2^ = 0.106		
CS	0.030	–0.290***				
RW1	0.110*	–0.061	–0.018			
RW2	–0.043	–0.015	–0.015	–0.244**		
RW3	0.136**	–0.065	–0.052	0.093	0.100	
RW4	0.110*	–0.024	0.063	0.060	0.015	–0.010
B. Female	*r* ^2^ = 0.021			*r* ^2^ = 0.098		
CS	–0.019	–0.236**				
RW1	0.037	–0.242*	–0.364*			
RW2	0.033	–0.137	–0.137	0.082		
RW3	–0.110*	0.057	–0.003	–0.017	–0.044	
RW4	–0.092*	0.015	–0.052	–0.045	–0.028	–0.140**

Randomization test: ^*^ *P* < 0.05; ^**^ *P* < 0.01; ^***^ *P* < 0.001.

**Figure 2:**
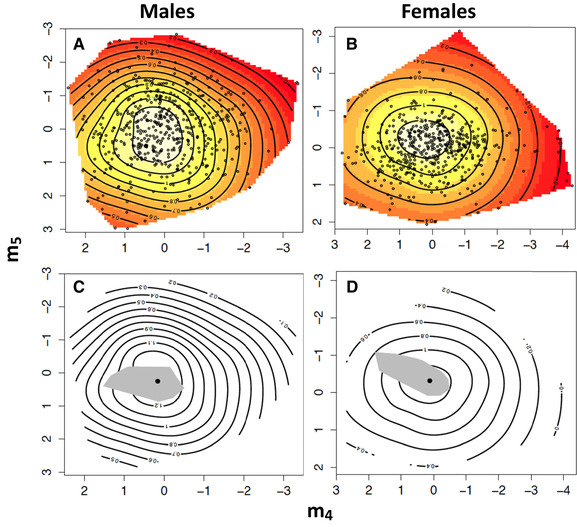
(A and B) Thin‐plate spline visualizations (contour view) of the two major axes of nonlinear selection (**m_4_** and **m_5_**) on the fitness surface for males and females, respectively. In each surface, white coloration represents regions of highest fitness, whereas red coloration represents regions of lowest fitness. Individual data points are provided as black circles on the surface. (C and D) Thin‐plate spline visualizations mapping the 95% confidence region of the global maxima (gray region) on the fitness surface for males and females. In each surface, the solid black dot represents the estimated location of the global maxima.

We conducted a canonical rotation of the **γ** matrices presented in Table 1 to locate the major dimensions of nonlinear sexual selection for male and female genital size and shape. The resulting **M** matrices of eigenvectors and their associated eigenvalues are presented in Table 2, panels A and B, respectively. In males, three of the five eigenvectors (**m_3_**–**m_5_**) had negative eigenvalues, whereas the remaining two eigenvectors (**m_1_** and **m_2_**) had positive eigenvalues (Table 2, panel A). However, there is only significant nonlinear selection operating on **m_4_** and **m_5_**, demonstrating that the fitness surface is best described as a multivariate peak in shape (Fig. 2A). There was also negative linear selection operating on **m_2_** that largely favors an increase in RW1 and RW4 (Table 2, panel A). In females, four of the five eigenvectors (**m_2_**–**m_5_**) had negative eigenvalues, whereas the remaining eigenvector (**m_1_**) had a positive eigenvalue (Table 2, panel B). As shown for males, significant nonlinear selection was only detected on **m_4_** and **m_5_**, indicating that the fitness surface for females is also best described as a multivariate peak in shape (Fig. 2B). There was also significant positive linear selection on **m_4_** that largely favors a reduction in RW4, and negative linear selection on **m_2_** that largely favors a reduction in RW3 (Table 2, panel B). The global maxima and their associated 95% CRs for **m_4_** and **m_5_** in males (**m_4_** = 0.222, **m_5_** = 0.275; Fig. 2C) and females (**m_4_** = 0.202, **m_5_** = –0.236; Fig. 2D) both exist within the distribution of phenotypic data sampled in Figures 2A and 2B, respectively. We can therefore be confident in formally defining the observed pattern of nonlinear selection in the sexes as multivariate stabilizing sexual selection (Mitchell‐Olds and Shaw 1987).

**Table 2:** The **M** matrix of eigenvectors from the canonical analysis of **γ** for successful mating in male and female *Tribolium castaneum*. The linear (**θ*_i_***) and quadratic (**λ*_i_***) gradient of sexual selection acting along each eigenvector (**m** *_i_*) are provided in the last two columns. The sign of **λ*_i_*** describes the form of quadratic selection acting along each eigenvector, with a positive **λ*_i_*** indicating disruptive selection and a negative **λ*_i_*** indicating stabilizing selection. The strength of selection acting along each eigenvector is given by |**λ*_i_***|

	M					Selection	
	CS	RW1	RW2	RW3	RW4	θ*_i_*	λ*_i_*
A. Male							
**m**_1_	0.120	0.229	–0.249	–0.927	–0.105	–0.098	0.149
**m**_2_	0.172	–0.654	–0.123	–0.024	–0.726	–0.144**	0.063
**m**_3_	–0.190	0.682	–0.136	0.253	–0.645	0.038	–0.077
**m**_4_	–0.024	–0.072	–0.951	0.210	0.214	0.084	–0.279***
**m**_5_	0.959	0.222	0.003	0.176	0.021	0.079	–0.317**
B. Female							
**m**_1_	0.299	0.093	–0.929	0.150	0.128	–0.061	0.149
**m**_2_	0.496	–0.430	0.232	0.718	–0.007	–0.096*	–0.009
**m**_3_	0.418	–0.450	0.071	–0.576	0.535	–0.008	–0.060
**m**_4_	0.375	–0.191	–0.071	–0.359	–0.830	0.099*	–0.175***
**m**_5_	–0.591	–0.753	–0.271	0.043	–0.089	–0.022	–0.610*

Randomization test: ^*^ *P* < 0.05, ^**^ *P* < 0.01, ^***^ *P* < 0.001.

**Figure 3:**
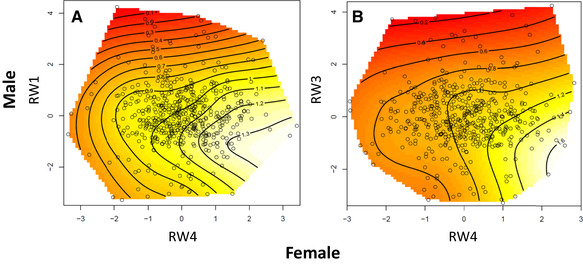
Thin‐plate spline visualizations (contour view) showing the two significant cases of negative correlational selection operating on the covariance between male and female genital shape: (A) RW1 in males and RW4 in females and (B) RW3 in males and RW4 in females. In both instances, white coloration represents regions of highest fitness, whereas red coloration represents regions of lowest fitness. Individual data points are provided as black circles on the surface.

Table 3 provides the interaction matrix of standardized correlational selection gradients for genital size and shape across the sexes. There was significant negative correlational selection on RW1 in males and RW4 in females (Table 3) and inspection of the thin‐plate spline (Fig. 3A) showed that fitness was highest at negative values of RW1 in males and positive values of RW4 in females (Fig. 3A). Consequently, the fitness of an interacting male and female beetle is highest when males have a short, wide aedeagus (Fig. 1A) and females have a narrower, tapering vaginal aperture and posterior elongation of the associated supportive structures (Fig. 1H). There was also significant negative correlational selection on RW3 in males and RW4 in females (Table 3) and inspection of the thin‐plate splines (Fig. 3B) showed that fitness was highest at negative values of RW3 in males and positive values of RW4 in females. As a result, the fitness of an interacting pair is highest when males have an anticlockwise twist to the anterior tip of the aedeagus (Fig. 1E) and females have a narrower, tapering vaginal aperture and posterior elongation of the supportive structures (Fig. 1H).

**Table 3:** Interaction matrix containing the standardized correlational selection gradients operating on the covariance between male and female genital size and shape

		Males				
		CS	RW1	RW2	RW3	RW4
Females	CS	0.062				
	RW1	0.092	0.016			
	RW2	–0.044	–0.029	–0.013		
	RW3	0.036	–0.046	–0.031	0.032	
	RW4	–0.025	–0.076^*^	0.007	–0.085*	0.031

Randomization test: ^*^ *P* < 0.05.

## Discussion

Explaining why the genital morphology of males is so highly divergent in species with internal fertilization has intrigued evolutionary biologists for decades (Hosken and Stockley 2004; Simmons 2014). Here, we show that male and female genital size and shape play an important role in the successful transfer of a spermatophore during mating in the red flour beetle (*T. castaneum*). This imposes strong sexual selection on male and female genital morphology that is multivariate stabilizing in form, being characterized by a well‐defined peak in fitness at intermediate values of genital size and shape in both sexes. We also found that sexual selection targeted the covariance between the sexes for specific aspects of genital shape, indicating that the interaction between male and female genitals also plays an important role in the successful transfer of a spermatophore during mating. Collectively, our work highlights the important yet often‐ignored role that female genital morphology plays in determining mating success (Ah‐King et al. 2014; Simmons 2014) and shows that these effects can occur independently, as well as through their interaction with male genital morphology. Moreover, our work cautions against the overly simplistic view that the sexual selection targeting genital morphology will always be directional in form (Arnqvist 1997).

Over two decades ago, Arnqvist (1997) provided a set of criteria to distinguish among the three major hypotheses of genital evolution for use in single species studies, with the form of selection targeting male genital morphology being an important criterion in this checklist (Arnqvist 1997). Our finding that the effects of male genital size and shape on mating success in *T. castaneum* generate a pattern of multivariate stabilizing sexual selection on this trait is in general agreement with Arnqvist's (1997) within‐species view of the lock‐and‐key hypothesis, and demonstrates a clear and important role for sexual selection in male genital evolution in this species. In insects, linear selection on male genital morphology (e.g., Cordoba‐Aguilar 1999, 2002, 2009; Arnqvist and Danielsson 1999, Danielsson and Askenmo 1999, Bertin and Fairbairn 2005; Wenninger and Averill 2006; Holwell et al. 2010; van Lieshout 2011; van Lieshout and Elgar 2011) appears more common than stabilizing selection (e.g., Tadler 1999; Simmons et al. 2009; Wojcieszek and Simmons 2012; House et al. 2016; Dougherty and Shuker 2016). This pattern also appears true more generally for selection on male sexual traits, although it should be noted that most experimental designs have more power to detect linear than nonlinear forms of selection (Higgins et al. 2009; Hunt et al. 2010). Interestingly, stabilizing sexual selection does appear common in a number of signaler‐receiver systems. For example, female mate choice exerts multivariate stabilizing selection on temporal and spectral components of the male advertisement call in grasshoppers (Butlin et al. 1985), field crickets (Brooks et al. 2005), and anurans (Gerhardt 1991; Polakow et al. 1995; Gerhardt and Brooks 2009). In these systems, the transmission of an acoustic signal is constrained by the biophysics of signal generation and emission, and how the sensory organs of the receiver perceive and process the signal (Endler and Basolo 1998). As the sensory system of the female can only detect call components within a narrow range, this produces a pattern of multivariate stabilizing selection acting on male call structure (Endler and Basolo 1998). Although the most parsimonious explanation for the pattern of multivariate stabilizing sexual selection we observe on male genital morphology in *T. castaneum* is the “mechanical fit” of genitals, we cannot rule out the possibility that a similar “sensory‐based” lock‐and‐key process does not exist. Indeed, Eberhard et al. (1998) has argued that male genitals are perceived by the female using tactile channels, with an intermediate male genital morphology being favored because they best stimulate the average female in the population (the “one‐size‐fits‐all” hypothesis). Distinguishing between mechanical and sensory lock‐and‐key processes, however, has proven difficult (Eberhard et al. 1998) and clearly more work is needed before this can be achieved in *T. castaneum*.

Although the evolution of male genital morphology has been the subject of intense research, female genitals have been relatively understudied (Ah‐King et al. 2014). This bias in genital research likely stems from the long‐held view that males play the dominant role in sex, and that female genitals are largely invariant (Ah‐King et al. 2014). This bias is likely to be further compounded by the fact that variation in male genital morphology is typically large across populations and species (Wojcieszek and Simmons 2012; Soto et al. 2013; Hosken et al. 2019) making them far easier to study. Our work, however, directly challenges these views by showing that female genital morphology in *T. castaneum* is far from invariant, and that the existing variation in this trait is also subject to strong multivariate stabilizing sexual selection. In fact, the nonlinear selection gradients describing the pattern of multivariate stabilizing sexual selection acting on female genital morphology were as strong as those reported for male genital morphology, further highlighting the equally important role that females play in determining the outcome of mating in this species. Although the major models of genital evolution do not provide any clear predictions regarding the strength and form of selection acting on female genitals, we believe that the pattern of sexual selection we document for female *T. castaneum* adds further support to an important role for a “lock‐and‐key” process in the evolution of genital morphology in this species. The operation of the “lock‐and‐key” process centers on the alignment of the male and female genitals during mating, with the optimal male genital morphology being the one that, on average, most closely aligns with the average female genital morphology in the population. As any deviation from this optimal morphology decreases the fit with the female genitals and reduces subsequent mating success, stabilizing selection is predicted to target male genital morphology (Arnqvist 1997). However, because the successful outcome of mating depends on both sexes, the poor alignment of genitals and the resulting reduction in mating success can also be caused by the female genitals deviating from the “average” in the population, which should also generate stabilizing selection on female genital morphology. Furthermore, as the “lock‐and‐key” process is based on the interaction between male and female genitals during mating, sexual selection should also target the covariance between these traits across the sexes—a finding that is also supported by our study. Collectively, our work highlights the value of formally estimating sexual selection on genital morphology in both sexes, and supports recent claims that our understanding of genital evolution will continue to be hampered until the persisting male bias in genital research is addressed (Ah‐King et al. 2014).

Although the strength and statistical significance of our estimated selection gradients indicate that stabilizing selection is the predominant form of selection targeting male and female genital morphology in *T. castaneum*, it is important to note that significant linear selection was also detected in both sexes for several aspects of genital shape (RW1, RW3, and RW4 in males and RW3 and RW4 in females) and also along the major eigenvectors of selection (**m_2_** in males and **m_2_** and **m_4_** in females). Exactly how these aspects of genital shape provide an advantage to males and females during the transfer of a spermatophore is currently unclear, although it is possible that it helps better position the male genitals or the ability of the female genitals to accommodate these structures during the transfer of a spermatophore (e.g., Werner and Simmons 2008). It is important to highlight, however, that because we only examined a single episode of selection (i.e., mating success), this is likely to provide an incomplete measure of all the linear selection targeting male and female genital morphology, and consequently the total sexual selection acting on these traits. This is particularly true given that a number of postcopulatory processes in insects, including sperm competition (e.g. Danielsson and Askenmo 1999; Wenninger and Averill 2006) and cryptic female choice (e.g., Cordoba‐Aguilar 1999), are known to impose significant linear selection on male genital morphology, although the contribution of female genitals to these episodes of selection remains largely unknown (Ah‐King et al. 2014). Consequently, the selection gradients we provide should only be interpreted within the context of mating success and not considered as estimates of total selection acting on male and female genital morphology.

Our work shows that male and female genital morphology not only has important independent effects on the successful transfer of a spermatophore during mating in *T. castaneum*, but also that the interaction between these traits influences mating success. That is, sexual selection targets male and female genital morphology directly, as well as indirectly via the covariance between these traits. To our knowledge, this is the first time such an approach has been used to study genital evolution, despite offering a novel means to assess how important the interaction of male and female genital morphology is to mating success. In *T. castaneum*, we found significant negative correlational selection gradients for two aspects of genital shape: RW4 in females with RW1 and RW3 in males. Biologically, this means that a mating pair will have a higher success in transferring a spermatophore when a male with either a short, wide aedeagus (RW1) or an anticlockwise twist to the anterior tip of the aedeagus (RW3) mates with a female having a narrower, tapering vaginal aperture and posterior elongation of the supportive structures (RW4). Explaining these relationships at this stage would be purely speculative, as isolating a mechanism requires functional studies (Ah‐King et al. 2014; Simmons 2014; Brennan and Prum 2015). Unfortunately, only a handful of such studies exist for insects (Ronn et al. 2007; Werner and Simmons 2008; Polak and Rashed 2010; Kahn et al. 2010; Hotzy et al. 2012). For example, Werner and Simmons (2008) used histology to show that three of the genital sclerites in male dung beetles (*Onthophagus taurus*) form a functionally integrated unit that generates the tubular‐shaped spermatophore and delivers its opening to the female's spermathecal duct, whereas a fourth serves as a holdfast device during mating. It is possible that a similar mechanical process enables a short, wide aedeagus in *T. castaneum* or one with an anticlockwise twist to the anterior tip to more efficiently deliver a spermatophore or better anchor the male when mating to a female with a narrower, tapering vaginal aperture with posterior elongation of the supportive structures. However, detailed functional studies are clearly needed to confirm this.

The pattern of sexual selection we document for male and female genital morphology in *T. castaneum* is likely to provide a number of important insights for genital evolution. First, strong linear and stabilizing selection is predicted to deplete the additive genetic variance of phenotypic traits (Lande 1979). This is especially true for male and female genital morphology in *T. castaneum*, where our estimated quadratic selection gradients were almost twice the median strength (**γ** = |0.16|) reported across studies based on mating success (Kingsolver et al. 2001). Indeed, where stabilizing selection has been demonstrated, it appears equally as strong, at least for insects (**γ** = –0.28, Tadler 1999; **γ** = –0.19 and –0.34, House et al. 2016; **γ** = –0.38, Dougherty and Shuker 2016). Yet, genital morphology is known to evolve rapidly (e.g., Simmons et al. 2009; House et al. 2013; Hopwood et al. 2016) and contain as much additive genetic variance as nongenital traits (e.g., Arnqvist and Thornhill 1998; House and Simmons 2005; Higgins et al. 2009; Kamimura and Iwase 2010). This suggests that mechanism(s) must exist to preserve the additive genetic variance of genital morphology, as has been argued more generally for sexual traits subject to strong selection (Kirkpatrick and Ryan 1991). Second, in a population subject to persistent stabilizing selection in the absence of frequency‐dependent selection, theory predicts that the population mean phenotype should evolve to match the peak in fitness (Simpson 1953; Lande 1979). Indeed, Estes and Arnold (2007) showed that a quantitative genetic model where the fitness optimum was able to evolve to the optimum within an adaptive zone with stable boundaries performed significantly better than five other competing models to explain the evolution of phenotypic means in an extensive database (Gingerich 2001). If the presence of stabilizing selection and the evolutionary response of the population mean is more common than generally appreciated for genitals, as might be expected under the lock‐and‐key hypothesis, it is relatively easy to envisage intra‐ and interspecific differences in genital morphology evolving as a result of variation in the location of the adaptive optima (Hansen 1997). This would certainly help explain the adaptive radiation in genital morphology frequently observed across natural (e.g., Wojcieszek and Simmons 2012; Heinen‐Kay and Langerhans 2013; Oneal and Knowles 2013) and experimental (e.g. Simmons et al. 2009; House et al. 2013; Hopwood et al. 2016) populations, as well as the extreme diversification in genitals observed across closely related species (e.g., Kuntner et al. 2016). Finally, theory predicts that correlational selection will generate a genetic correlation between the two traits by creating linkage disequilibrium (Lande 1984) or by favoring pleiotropic mutations (Lande 1980), although these mechanisms are not strictly required if correlational selection is strong and persistent (Sinervo and Svensson 2002). Although we do not know of any formal models examining whether correlational selection can also generate intersexual genetic correlations, there is empirical evidence showing that artificial selection on the covariance between male and female traits can dramatically alter the strength of the intersexual genetic correlation between these traits (Delph et al. 2011; Stewart and Rice 2018). It is therefore possible that the correlational selection we document on the covariance between aspects of genital shape in male and female *T. castaneum* will also promote these trait being genetically correlated across the sexes. Ultimately, this will facilitate the co‐evolution of male and female genital shape in *T. castaneum* (Lande 1980), and may explain why the sexual co‐evolution of genitals has been so widely documented in both experimental evolution and comparative studies (reviewed in Brennan and Prum 2015).

In conclusion, our study shows that both male and female genital morphology is subject to strong multivariate stabilizing sexual selection in *T. castaneum*, but that sexual selection also targets the covariance between the sexes for aspects of genital shape, indicating that how the genitals interact during mating is also important to the successful transfer of a spermatophore in this species. Both findings provide empirical support for the within species “lock‐and‐key” hypothesis of genital evolution (Arnqvist 1997), although we cannot determine at this stage whether this process is driven by a mechanical or sensory‐based interaction (or both) during mating. The pattern of sexual selection we document for male and female genital morphology in *T. castaneum* is likely to have a number of important implications for genital evolution, including explaining the adaptive radiation of genitals across populations and the diversification of closely related species, as well as the co‐evolution of male and female genital morphology.

Associate Editor: K. McGuigan

Handling Editor: M. Servedio

## Supporting information


**Figure S1**. To facilitate GM measurement of the male aedeagus, a total of 5 type 2 landmarks that are discrete and could be consistently identified were located (( ) ‐ 1, 6, 10, 14 and 19) and represent points at the endpoint of the aedaegus (points 1, 10 and 19) and at the minimum curvature of the bulge at the tip of the aedaegus (points 6 and 14) (Zelditch et al., 2014).
**Figure S2**. To facilitate GM measurement of the female vagina and supporting structures, 18 type 2 landmarks that could be consistently identified ( ( ) ‐ 1, 5, 6, 7, 8, 13, 14, 16, 18, 19, 21, 23, 24, 29, 30, 31, 32 and 36) and another 18 sliding semi‐landmarks were placed along the female genital outline using the programs described above (Figure S1).
**Table S1**. Repeatability estimates and 95% confidence intervals (CIs) for centroid size (CS) and the first four relative warp (RW) scores describing genital shape in male and female *T. castaneum*.
**Table S2**. The vector of standardized linear selection gradients (***β***) and the matrix of quadratic and correlational selection gradients (**γ**) for successful mating in male and female *Tribolium castaneum*.

## References

[evo13912-bib-0001] Ah‐King, M. , A. B. Barron , and M. E. Herberstein . 2014 Genital *evolution*: why are females still understudied? PLoS Biol. 12:e1001851. 24802812 10.1371/journal.pbio.1001851 PMC4011675

[evo13912-bib-0002] Anderson, C. M. , and R. B. Langerhans . 2015 Origins of female genital diversity: predation risk and lock‐and‐key explain rapid divergence during an adaptive radiation. Evolution 69:2452–2467. 26259062 10.1111/evo.12748

[evo13912-bib-0003] Arnqvist, G. 1997 The evolution of animal genitalia: distinguishing between hypotheses by single species studies. Biol. J. Linn. Soc. 60:365–379.

[evo13912-bib-0004] Arnqvist, G. , and R. Thornhill . 1998 Evolution of animal genitalia: patterns of phenotypic and genotypic variation and condition dependence of genital and non‐genital morphology in water strider (Heteroptera: Gerridae: Insecta). Genet. Res. 71:193–212.

[evo13912-bib-0005] Arnqvist, G. , and I. Danielsson . 1999 Copulatory behaviour, genital morphology, and male fertilization success in water striders. Evolution 53:147–156. 28565197 10.1111/j.1558-5646.1999.tb05340.x

[evo13912-bib-0006] Arnqvist, G. , R. Thornhill , and L. Rowe . 1997 Evolution of animal genitalia: morphological correlates of fitness components in a water strider. J. Evol. Biol. 10:613–640.

[evo13912-bib-0007] Attia, F. A. , and T. Tregenza . 2004 Divergence revealed by population crosses in the red flour beetle *Tribolium castaneum* . Evol. Ecol. Res. 6:927–935.

[evo13912-bib-0008] Bentsen, C. L. , J. Hunt , M. D. Jennions , and R. Brooks . 2006 Complex multivariate sexual selection on male acoustic signalling in a wild population of *Teleogryllus commodus* . Am. Nat. 167:E102–E116. 16670989 10.1086/501376

[evo13912-bib-0009] Bertin, A. , and D. J. Fairbairn . 2005 One tool, many uses: precopulatory sexual selection on genital morphology in *Aquarius remigis* . J. Evol. Biol. 18:949–961. 16033567 10.1111/j.1420-9101.2005.00913.x

[evo13912-bib-0010] Bisgaard, S. , and B. Ankenman . 1996 Standard errors for the eigenvalues in second‐order response surface models. Technometrics 38:238–246.

[evo13912-bib-0011] Bloch Qazi, M. C. , J. T. Herbeck , and S. M. Lewis . 1996 Mechanisms of sperm transfer and storage in the red flour beetle (Coleoptera: Tenebrionidae). Ann. Entomol. Soc. Am. 89:892–897.

[evo13912-bib-0012] Blows, M. W. , and R. Brooks . 2003 Measuring nonlinear selection. Am. Nat. 162:815–820. 14737718 10.1086/378905

[evo13912-bib-0013] Blows, M. W. , and A. A. Hoffmann . 2005 A reassessment of genetic limits to evolutionary change. Ecology 86:1371–1384.

[evo13912-bib-0014] Blows, M. W. , R. Brooks , and P. G. Kraft . 2003 Exploring complex fitness surfaces: multiple ornamentation and polymorphism in male guppies. Evolution 57:1622–1630. 12940366 10.1111/j.0014-3820.2003.tb00369.x

[evo13912-bib-0015] Booksmythe, I. , H. M.L. , S. Keogh , and M. D. Jennions . 2016 Fitness consequences of artificial selection on relative male genital size. Nat. Comm. 7:11597. 10.1038/ncomms11597 PMC4873965 27188478

[evo13912-bib-0016] Brennan, P. L. R. , and R. O. Prum . 2015 Mechanisms and evidence of genital coevolution: the roles of natural selection, mate choice, and sexual conflict. Cold Spring Harb. Persect. Biol. 7:a017749. 10.1101/cshperspect.a017749 PMC4484975 26134314

[evo13912-bib-0017] Brennan, P. L. R. , C. Clark , and R. O. Prum . 2010 Explosive eversion and functional morphology of the duck penis supports sexual conflict in waterfowl genitalia. Proc. R. Soc. B 277:1309–1314. 10.1098/rspb.2009.2139 PMC2871948 20031991

[evo13912-bib-0018] Brennan, P. L. R. , I. Gereg , M. Goodman , D. Feng , and R. O. Prum . 2017 Evidence of phenotypic placiticity of penis morphology and delayed reproductive maturation in response to male competition in waterfowl. Auk 134:882–893.

[evo13912-bib-0019] Brooks, R. , J. Hunt , M. W. Blows , M. J. Smith , L. F. Bussière , and M. D. Jennions . 2005 Experimental evidence for multivariate stabilizing sexual selection. Evolution 59:871–880. 15926696

[evo13912-bib-0020] Butlin, R. K. , G. M. Hewitt , and S. F. Webb . 1985 Sexual selection for intermediate optimum in *Chorthippus brunneus* (Orthoptera: Acrididae). Anim. Behav. 33:1281–1292.

[evo13912-bib-0021] Chenoweth, S. F. , and M. W. Blows . 2005 Contrasting mutual selection on homologous signal traits in *Drosophila serrata* . Am. Nat. 165:281–289. 15729657 10.1086/427271

[evo13912-bib-0022] Cordoba‐Aguilar, A. 1999 Male copulatory sensory stimulation induces female ejection of rival sperm in a damselfly. Proc. R. Soc. Lond. B 266:779–784.

[evo13912-bib-0023] Cordoba‐Aguilar, A. 2002 Sensory trap as the mechanism of sexual selection in a damselfly genitalic trait (Insecta: Calopterygidae). Am. Nat. 160:594–601. 18707510 10.1086/342819

[evo13912-bib-0024] Cordoba‐Aguilar, A. 2009 Seasonal variation in genital and body size, sperm displacement ability, female mating rate, and male harassment in two calopterygid damselfies (Odonata: Calopterygidae). Biol. J. Linn. Soc. 96:815–829.

[evo13912-bib-0025] Danielsson, I. , and C. Askenmo . 1999 Male genital traits and mating interval affect male fertilization success in the water strider. Behav. Ecol. Socio. 46:149–156.

[evo13912-bib-0026] del Castillo, E. , J. Hunt , and J. Rapkin . 2016 OptimaRegion: confidence regions for Optima. R package version 0.2. Available via https://CRAN.R-project.org/package=OptimaRegion.

[evo13912-bib-0027] Delph, L. F. , J. C. Steven , I. A. Anderson , C. R. Herlihy , and E. D. Brodie III . 2011 Elimination of a genetic correlation between the sexes via artificial correlational selection. Evolution 65:2872–2880. 21967428 10.1111/j.1558-5646.2011.01350.x

[evo13912-bib-0028] Dougherty, L. R. , and D. M. Shuker . 2016 Variation in pre‐ and post‐copulatory sexual selection on male genital size in two species of lygaeid bug. Behav. Ecol. Socio. 70:625–637. 10.1007/s00265-016-2082-6 PMC4788681 27069302

[evo13912-bib-0029] Dufour, L. 1844 Anatomie générale des diptères. Ann. Sci. Nat. 1:244–264.

[evo13912-bib-0030] Eberhard, W. G. 2001 Genitalic behavior in *Hybosciara gigantea* (Diptera: Sciaridae) and the evolution of spcies‐specific genitalia. J. Kans. Entomol. Soc. 74:1–9.

[evo13912-bib-0031] Eberhard, W. G. , B. A. Huber , S. Rafael Lucas Rodriguez , R. D. Briceno , I. Salas , and V. Rodriguez . 1998 One size fits all? Relationships between the size and degree of variation in genitalia and other body parts in twenty species of insects and spiders. Evolution 52:415–431. 28568329 10.1111/j.1558-5646.1998.tb01642.x

[evo13912-bib-0032] Endler, J. A. , and A. L. Basolo . 1998 Sensory ecology, receiver biases and sexual selection. Trends Ecol. Evol. 13:415–420. 21238370 10.1016/s0169-5347(98)01471-2

[evo13912-bib-0033] Estes, S. , and S. J. Arnold . 2007 Resolving the paradox of stasis: Models with stabilizing selection explain evolutionary divergence on all timescales. Am. Nat. 169:227–244. 17211806 10.1086/510633

[evo13912-bib-0034] Fedina, T. Y. , and S. M. Lewis . 2008 An integrative view of sexual selection in *Tribolium* flour beetles. Biol. Rev. 83:151–171. 18429767 10.1111/j.1469-185X.2008.00037.x

[evo13912-bib-0035] Foellmer, M. W. 2008 Broken genitals function as mating plugs and affect sex ratios in the orb‐web spider *Argiope aurantia* . Evol. Ecol. Res. 10:449–462.

[evo13912-bib-0036] Gerhardt, H. C. 1991 Female mate choice in treefrogs: static and dynamic acoustic criteria. Anim. Behav. 42:615–635.

[evo13912-bib-0037] Gerhardt, H. C. , and R. Brooks . 2009 Experimental analysis of multivariate female choice in gray treefrogs (*Hyla versicolor*): evidence for directional and stabilizing selection. Evolution 63:2504–2512. 19500145 10.1111/j.1558-5646.2009.00746.x PMC2763017

[evo13912-bib-0038] Gingerich, P. D. 2001 Rates of evolution on the time scale of the evolutionary process Pp. 127–144 *in* Hendry A. P. and Kinnison M. T., eds. Contemporary microevolution: rate, pattern, and process. Kluwer Academic Publishers, Dordrecht, the Netherlands.

[evo13912-bib-0039] Green, P. J. , and B. W. Silverman . 1994 Nonparametric regression and generalised linear models. Chapman and Hall, Lond.

[evo13912-bib-0040] Gutierrez, B. L. , N. Macleod , and G. D. Edgecombe . 2011 Detecting taxonomic signal in an under‐utilised character system: geometric morphometrics of the forcipular coxae of Scutigeromorpha (Chilopoda). ZooKeys 66:49–66. 10.3897/zookeys.156.1997 PMC3253570 22303095

[evo13912-bib-0041] Hansen, T. F. 1997 Stabilizing selection and the comparative analysis of adaptation. Evolution 51:1341–1351. 28568616 10.1111/j.1558-5646.1997.tb01457.x

[evo13912-bib-0042] Heinen‐Kay, J. L. , and R. B. Langerhans . 2013 Predation‐associated divergence of male genital morphology in a livebearing fish. J. Evol. Biol. 26:2135–2146. 23980714 10.1111/jeb.12229

[evo13912-bib-0043] Hersch, E. I. , and P. C. Phillips . 2004 Power and potential bias in field studies of natural selection. Evolution 58:479–485. 15119432

[evo13912-bib-0044] Higgins, S. L. , D. J. Hosken , and N. Wedell . 2009 Phenotypic and genetic variation in male genitalia in the seedbug, *Lygaeus equestris* (Heteroptera). Biol. J. Linn. Soc. 98:400–405.

[evo13912-bib-0045] Holwell, G. I. , C. Winnick , T. Tregenza , and M. E. Herberstein . 2010 Genital shape correlates with sperm transfer success in the praying mantis *Ciulfina klassi* (Insecta: Mantodea). Behav. Ecol. Socio. 64:617–625.

[evo13912-bib-0046] Hopwood, P. E. , M. L. Head , E. J. Jordan , M. J. Carter , E. Davey , A. J. Moore , and N. J. Royle . 2016 Selection on an antagonistic behavioral trait can drive rapid genital coevolution in the burying beetle, *Nicrophorus vespilloides* . Evolution 70:1180–1188. 27144373 10.1111/evo.12938 PMC5089618

[evo13912-bib-0047] Hosken, D. J. , and P. Stockley . 2004 Sexual selection and genital evolution. Trends Ecol. Evol. 19:87–93. 16701234 10.1016/j.tree.2003.11.012

[evo13912-bib-0048] Hotzy, C. , M. Polak , J. L. Ronn , and G. Arnqvist . 2012 Phenotypic engineering unveils the function of genital morphology. Current. Biol. 22:2258–2261. 10.1016/j.cub.2012.10.009 23103188

[evo13912-bib-0049] House, C. M. , and L. W. Simmons . 2003 Genital morphology and fertilization success in the dung beetle *Onthophagus taurus*: an example of sexually selected male genitalia. Proc. R. Soc. Lond. B 270:447–455. 10.1098/rspb.2002.2266 PMC1691274 12641898

[evo13912-bib-0050] House, C. M. , and L. W. Simmons 2005 The evolution of male genitalia: patterns of genetic variation and covariation in the genital sclerites of the dung beetle *Onthophagus taurus* . J. Evol. Biol. 18:1281–1292. 16135123 10.1111/j.1420-9101.2005.00926.x

[evo13912-bib-0051] House, C. M. , Z. Lewis , D. J. Hodgson , N. Wedell , M. D. Sharma , J. Hunt , and D. J. Hosken . 2013 Sexual and natural selection both influence male genital evolution. PLoS ONE 8:e63807. 23717488 10.1371/journal.pone.0063807 PMC3661765

[evo13912-bib-0052] House, C. M. , M. D. Sharma , K. Okada , and D. J. Hosken . 2016 Pre and post‐copulatory selection favour similar genital phenotypes in the male broad horned beetle. Integr. Comp. Biol. 56:682–693. 27371390 10.1093/icb/icw079 PMC5035384

[evo13912-bib-0053] Hosken, D. J. , C. R. Archer , C. M. House , and D. J. Hosken . 2019 Penis evolution across species: divergence and diversity. Nat. Rev. Urol. 16:98–106. 30397329 10.1038/s41585-018-0112-z

[evo13912-bib-0054] Hunt, J. , C. J. Breuker , J. A. Sadowski , and A. J. Moore . 2010 Male‐male competition, female mate choice and their interaction: determining total sexual selection. J. Evol. Biol. 22:13–26. 10.1111/j.1420-9101.2008.01633.x 19120810

[evo13912-bib-0055] Johnson, S. G. 2014 The NLopt nonlinear optimization package. Available via http://ab-initio.mit.edu/nlopt.

[evo13912-bib-0056] Kahn, A. T. , B. Mautz , and M. D. Jennions . 2010 Females prefer to associate with males with longer intromittent organs in mosquitofish. Biol. Lett. 6:55–58. 19755529 10.1098/rsbl.2009.0637 PMC2817265

[evo13912-bib-0057] Kamimura, Y. , and R. Iwase . 2010 Evolutionary genetics of genital sie and lateral asymmetry in the earwig *Euborellia plebeja* (Dermaptera: Anisolabididae). Biol. J. Linn. Soc. 101:103–112.

[evo13912-bib-0058] King, R. B. , R. C. Jadin , M. Grue , and H. D. Walley . 2009 Behavioural correlates with hemipenis morphology in new world natricine snakes. Biol. J. Linn. Soc. 98:110–120.

[evo13912-bib-0059] Kingsolver, J. G. , H. E. Hoekstra , J. M. Hoekstra , D. Berrigan , S. N. Vignieri , C. E. Hill , A. Hoang , P. Gibert , and P. Beerli . 2001 The strength of phenotypic selection in natural populations. Am. Nat. 157:245–261. 18707288 10.1086/319193

[evo13912-bib-0060] Kirkpatrick, M. , and M. J. Ryan . 1991 The evolution of mating preferences and the paradox of the lek. Nature 350:33–38.

[evo13912-bib-0061] Klaczo, J. , T. Ingram , and J. Losos . 2015 Genitals evolve faster than other traits in *Anolis* lizards. J. Zool. 295:44–48.

[evo13912-bib-0062] Kuntner, M. , R.‐C. Cheng , S. Kralj‐Fišer , C. Liao , J. M. Schneider , and M. A. Elgar . 2016 The evolution of genital complexity and mating rates in sexually size dimorphic spiders. BMC Evol. Biol 16:242. 27829358 10.1186/s12862-016-0821-y PMC5103378

[evo13912-bib-0063] Lande, R. 1979 Quantitative genetical analysis of multivariate evolution, applied to brain: body size allometry. Evolution 33:402–416. 28568194 10.1111/j.1558-5646.1979.tb04694.x

[evo13912-bib-0064] Lande, R. 1980 The genetic covariance between characters maintained by pleiotropic mutations. Genetics 94:203–215. 17248993 10.1093/genetics/94.1.203 PMC1214134

[evo13912-bib-0065] Lande, R. 1984 The genetic correlation between characters maintained by selection, linkage and inbreeding. Genet. Res. Camb. 44:309–320. 10.1017/s0016672300026549 6530140

[evo13912-bib-0066] Lande, R. , and S. J. Arnold . 1983 The measurement of selection on correlated characters. Evolution 37:1210–1226. 28556011 10.1111/j.1558-5646.1983.tb00236.x

[evo13912-bib-0067] Langerhans, R. B. , C. A. Layman , and T. J. DeWitt . 2005 Male genital size reflects a tradeoff between attracting mates and avoiding predators in two live‐bearing fish species. Proc. Natl. Acad. Sci. 102:7618–7623. 15894618 10.1073/pnas.0500935102 PMC1140428

[evo13912-bib-0068] Lewis, S. M. , and J. Iannini . 1995 Fitness consequences of differences in male mating behaviour in relation to female reproductive status in flour beetles. Anim. Behav. 50:1157–1160.

[evo13912-bib-0069] Manly, B. F. J. 1997 Randomization, bootstrap and Monte Carlo methods in biology. Chapman and Hall, Lond.

[evo13912-bib-0070] Mayr, E. 1963 Animal species and evolution. Harvard Univ. Press, Cambridge, MA.

[evo13912-bib-0071] Mitchell‐Olds, T. , and R. G. Shaw . 1987 Analysis of natural selection: statistical inference and biological interpretation. Evolution 41:1149–1161. 28563617 10.1111/j.1558-5646.1987.tb02457.x

[evo13912-bib-0072] Oneal, E. , and L. L. Knowles . 2013 Ecological selection as the cause and sexual differentiation as the consequence of species divergence. Proc. R. Soc. B 280:20122236. 10.1098/rspb.2012.2236 PMC3574439 23173206

[evo13912-bib-0073] Pai, A. , B. Lauren , and Y. Guiyun . 2005 Female multiple mating for fertility assurance in red flour beetles (*Tribolium casteneum*). Can. J. Zool. 83:913–919.

[evo13912-bib-0074] Peterson, J. , S. Cahya , and E. Del Castillo . 2002 A general approach to confidence regions for optimal factor levels of response surfaces. Biometrics 58:422–431. 12071416 10.1111/j.0006-341x.2002.00422.x

[evo13912-bib-0075] Phillips, P. C. , and S. J. Arnold . 1989 Visualizing multivariate selection. Evolution 43:1209–1222. 28564514 10.1111/j.1558-5646.1989.tb02569.x

[evo13912-bib-0076] Polak, M. , and A. Rashed . 2010 Microscale laser surgery reveals adaptive function of male intromittent genitalia. Proc. R. Soc. B 277:1371–1376. 10.1098/rspb.2009.1720 PMC2871932 20053645

[evo13912-bib-0077] Polakow, D. A. , P. Y. Backwell , N. Caithness , and M. D. Jennions . 1995 Stabilizing or directional selection in signalling systems: investigations in a population of painted reed frogs, *Hyperolius marmoratus* . S. Afr. J. Sci. 91:270–273.

[evo13912-bib-0078] Ramm, S. A. 2007 Sexual selection and genital evolution in mammals: A phylogenetic analysis of baculum length. Am. Nat. 169:360–369. 17238128 10.1086/510688

[evo13912-bib-0079] Reynolds, R. J. , D. K. Childers , and N. M. Pajewski . 2010 The distribution and hypothesis testing of eigenvalues from the canonical analysis of the gamma matrix of quadratic and correlation selection gradients. Evolution 64:1076–1085. 19863584 10.1111/j.1558-5646.2009.00874.x PMC2857515

[evo13912-bib-0080] Ronn, J. , M. Katvala , and G. Arnqvist . 2007 Coevolution between harmful male genitalia and female resistance in seed beetles. Proc. Natl. Acad. Sci. 104:10921–10925. 17573531 10.1073/pnas.0701170104 PMC1904142

[evo13912-bib-0081] Simmons, L. W. 2014 Sexual selection and genital evolution. Austral Entomol. 53:1–17.

[evo13912-bib-0082] Simmons, L. W. , C. M. House , J. Hunt , and F. Garcia‐Gonzalez . 2009 Evolutionary response to sexual selection in male genital morphology. Current. Biol. 19:1442–1446. 10.1016/j.cub.2009.06.056 19664925

[evo13912-bib-0083] Simpson, G. G. 1953 The major features of evolution. Columbia Univ. Press, Garden City, NY.

[evo13912-bib-0084] Sinervo, B. , and E. Svensson . 2002 Correlational selection and the evolution of genomic architecture. Heredity 89:329–338. 12399990 10.1038/sj.hdy.6800148

[evo13912-bib-0085] Soto, I. M. , V. P. Carreira , E. M. Soto , F. Márquex , P. Lipko , and E. Hasson . 2013 Rapid divergent evolution of male genitalia among populations of *Drosophila buzzatii* . Evol. Biol. 40:395–407.

[evo13912-bib-0086] Steiger, S. , G. D. Ower , J. Stökl , C. Mitchell , J. Hunt , and S. K. Sakaluk . 2013 Sexual selection on cuticular hydrocarbons of male sagebrush crickets in the wild. Proc. R. Soc. B 280:20132353. 10.1098/rspb.2013.2353 PMC3826231 24197415

[evo13912-bib-0087] Stewart, A. D. , and W. R. Rice . 2018 Arrest of sex‐specific adaptation during the evolution of sexual dimorphism in *Drosophila* . Nat. Ecol. Evol. 2:1507–1513. 30061561 10.1038/s41559-018-0613-4

[evo13912-bib-0088] Stinchcombe, J. R. , A. F. Agrawal , P. A. Hohenlohe , S. J. Arnold , and M. W. Blows . 2008 Estimating non‐linear selection gradients using quadratic regression coefficients: double or nothing? Evolution 62:2435–2440. 18616573 10.1111/j.1558-5646.2008.00449.x

[evo13912-bib-0089] Stockley, P. 2002 Sperm competition risk and male genital anatomy: comparative evidence for reduced duration of female sexual receptivity in primates with penile spines. Evol. Ecol. 16:123–137.

[evo13912-bib-0090] Tadler, A. 1999 Selection of a conspicuous male genitalic trait in the seedbug *Lygaeus simulans* . Proc. R. Soc. Lond. B 266:1773–1777.

[evo13912-bib-0091] Tyler, F. , and T. Tregenza . 2012 Why do so many flour beetle copulations fail? Entomol. Exp. Appl. 146:199–206.

[evo13912-bib-0092] Van Lieshout, E. 2011 Male genital length and mating status differentially affect mating behaviour in an earwig. Behav. Ecol. Socio. 65:149–156.

[evo13912-bib-0093] Van Lieshout, E. , and M. A. Elgar . 2011 Longer exaggerated male genitalia confer defensive sperm‐competition benefits in an earwig. Evol. Ecol. 25:351–362.

[evo13912-bib-0094] Wenninger, E. J. , and A. L. Averill . 2006 Influence of body and genital morphology on relative male fertilization success in oriental beetle. Behav. Ecol. 17:656–663.

[evo13912-bib-0095] Werner, M. , and L. W. Simmons . 2008 The evolution of male genitalia: functional integration of genital sclerites in the dung beetle *Onthophagus taurus* . Biol. J. Linn. Soc. 98:257–266.

[evo13912-bib-0096] Wojcieszek, J. M. , and L. W. Simmons . 2012 Evidence for stabilizing selection and slow divergent evolution of male genitalia in a millipede (*Antichiropus variabilis*). Evolution 66:1138–1153. 22486694 10.1111/j.1558-5646.2011.01509.x

[evo13912-bib-0097] Wojcieszek, J. M. 2013 Divergence in genital morphology may contribute to mechanical reproductive isolation in a millipede. Ecol. Evol. 3:334–343. 23467632 10.1002/ece3.466 PMC3586643

[evo13912-bib-0098] Yeh, A. B. , and K. Singh . 1997 Balanced confidence regions based on Tukey's depth and the bootstrap. J. R. Statist. Soc. B 59:639–652.

[evo13912-bib-0099] Ypma, J. 2014 Introduction to nloptr: an R interface to NLopt. Available via https://cran.r-project.org/web/packages/nloptr/.

