# Supplementary material for: Multivariate stabilizing sexual selection and the evolution of male and female genital morphology in the red flour beetle
Source: Evolution. 2020 Jan 23;74(5):883–96. doi: 10.1111/evo.13912 (PMC7317928; doi:10.1111/evo.13912)
Supplement: Supplementary file 1 — Figure S1. To facilitate GM measurement of the male aedeagus, a total of 5 type 2 landmarks that are discrete and could be consistently identified were located (( ) ‐ 1, 6, 10, 14 and 19) and represent points at the endpoint of the aedaegus (points 1, 10 and 19) and at the minimum curvature of the bulge at the tip of the aedaegus (points 6 and 14) (Zelditch et al., 2014). Figure S2. To facilitate GM measurement of the female vagina and supporting structures, 18 type 2 landmarks that could be consistently identified ( ( ) ‐ 1, 5, 6, 7, 8, 13, 14, 16, 18, 19, 21, 23, 24, 29, 30, 31, 32 and 36) and another 18 sliding semi‐landmarks were placed along the female genital outline using the programs described above (Figure S1). Table S1. Repeatability estimates and 95% confidence intervals (CIs) for centroid size (CS) and the first four relative warp (RW) scores describing genital shape in male and female T. castaneum. Table S2. The vector of standardized linear selection gradients (β) and the matrix of quadratic and correlational selection gradients (γ) for successful mating in male and female Tribolium castaneum. [file EVO-74-883-s001.docx]

**Online Supplement**

**Figure S1.** To facilitate GM measurement of the male aedeagus, a total of 5 type 2 landmarks that are discrete and could be consistently identified were located (( ) - 1, 6, 10, 14 and 19) and represent points at the endpoint of the aedaegus (points 1, 10 and 19) and at the minimum curvature of the bulge at the tip of the aedaegus (points 6 and 14) (Zelditch et al., 2014). We also included an additional 14 sliding semi-landmarks along the aedeagus outline to capture information about the curvature of the male aedeagus using the TPSUTIL (version 1.46) and TPSDig (version 2.14) programs (Rolf 2009). The Cartesian coordinates of these landmarks were extracted and normalized for position, orientation and scale (generalized least squares superimposition) to eliminate non-shape variation using the tpsRELW (version 1.46) program (Rohlf 2008). We estimated the overall size of the aedeagus from the centroid size calculated as the square root of the sum of squared distances of the landmarks from the centroid (Cardini 2012). Relative warps that are a principal components analysis of partial warps (Adams et al. 2009) were also calculated using tpsRELW (version 1.46; Rohlf 2008). Finally, changes in the shape of the aedeagus were visualized as shape deformations of thin plate splines in TPSRelw (version 1.46; Rohlf 2008).


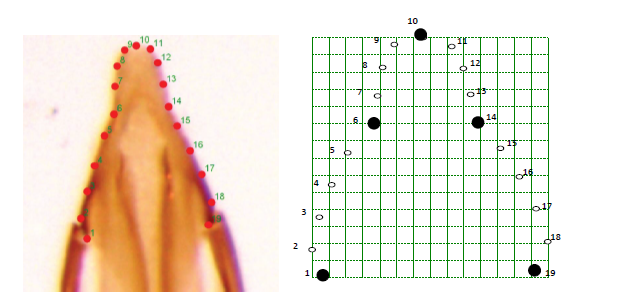


**Figure S2.** To facilitate GM measurement of the female vagina and supporting structures, 18 type 2 landmarks that could be consistently identified ( ( ) - 1, 5, 6, 7, 8, 13, 14, 16, 18, 19, 21, 23, 24, 29, 30, 31, 32 and 36) and another 18 sliding semi-landmarks were placed along the female genital outline using the programs described above (Figure S1). Female genital centroid size and relative warp (RW) scores were calculated and vagina shape changes were visualized as above (Figure S1).


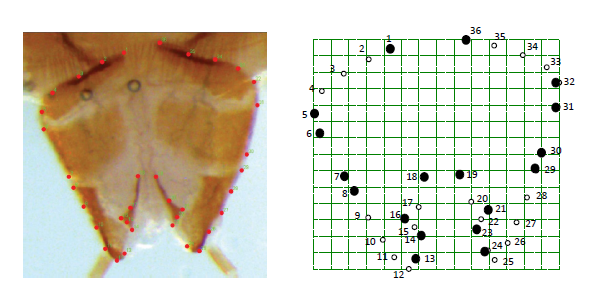


**Table S1.** Repeatability estimates and 95% confidence intervals (CIs) for centroid size (CS) and the first four relative warp (RW) scores describing genital shape in male and female *T. castaneum*. We measured the repeatability of digitization of two images of the same genitalia for a subset of 25 experimental males and females sampled from the parental population. The male and female genitalia both consist of thin membranous tissue, so it is not possible to re-mount specimens and test the repeatability of mounting. Reapeatbility estimates and 95% CIs were estimated using the R code provided in Wolak et al. (2012). All of our measurements of male and female genital morphology were repeatable.

| Sex | Trait | Repeatability | 95% CIs |
| --- | --- | --- | --- |
| Male | CS | 0.953 | 0.916, 0.989 |
|  | RW1 | 0.936 | 0.887, 0.985 |
|  | RW2 | 0.777 | 0.619, 0.933 |
|  | RW3 | 0.745 | 0.568, 0.921 |
|  | RW4 | 0.744 | 0.567, 0.921 |
| Female | CS | 0.993 | 0.988, 0.998 |
|  | RW1 | 0.992 | 0.994, 0.999 |
|  | RW2 | 0.963 | 0.934, 0.991 |
|  | RW3 | 0.988 | 0.978, 0.997 |
|  | RW4 | 0.940 | 0.895, 0.986 |

**Table S2.** The vector of standardized linear selection gradients (***β***) and the matrix of quadratic and correlational selection gradients (**γ**) for successful matings in male and female *Tribolium castaneum*. These selection estimates were derived from a full model where male and female genital measures plus their interaction were all included in the model. That is, estimates of selection on male genitals are controlling for the effects of variation in female genitals (and *vice versa* for female genitals). The ♂ and ♀ subscripts refer to male and female genital measures, respectively. The section of the table highlighted in grey represents the interaction matrix provided in Table 3. The *r*^2^ estimates for the linear and second order quadratic models were 0.050 and 0.206, respectively. Randomization test: * *P* < 0.05, ** *P* < 0.01, *** *P* < 0.001.

|  |  | **γ** | | | | | | | | | |
| --- | --- | --- | --- | --- | --- | --- | --- | --- | --- | --- | --- |
|  | ***β*** | CS_♂_ | RW1_♂_ | RW2_♂_ | RW3_♂_ | RW4_♂_ | CS_♀_ | RW1_♀_ | RW2_♀_ | RW3_♀_ | RW4_♀_ |
| CS_♂_ | 0.032 | -0.270** |  |  |  |  |  |  |  |  |  |
| RW1_♂_ | 0.108* | -0.082 | 0.002 |  |  |  |  |  |  |  |  |
| RW2_♂_ | -0.045 | -0.006 | 0.014 | -0.272** |  |  |  |  |  |  |  |
| RW3_♂_ | 0.138* | -0.027 | -0.073 | 0.068 | 0.112 |  |  |  |  |  |  |
| RW4_♂_ | 0.103* | -0.022 | 0.054 | 0.07 | 0.042 | -0.026 |  |  |  |  |  |
| CS_♀_ | -0.038 | 0.062 |  |  |  |  | -0.240** |  |  |  |  |
| RW1_♀_ | 0.004 | 0.092 | 0.016 |  |  |  | -0.208* | -0.312* |  |  |  |
| RW2_♀_ | 0.022 | -0.044 | -0.029 | -0.013 |  |  | -0.101 | -0.107 | 0.048 |  |  |
| RW3_♀_ | -0.113* | 0.036 | -0.046 | -0.031 | 0.032 |  | 0.048 | 0.013 | -0.04 | -0.008 |  |
| RW4_♀_ | -0.098* | -0.025 | -0.076* | 0.007 | -0.085* | 0.031 | 0.063 | 0.037 | -0.062 | 0.011 | -0.132* |

**References**

Rohlf, F.J. 2006. *tpsUtil, file utility program*, Version 1.38. Stony Brook NY: Department of Ecology and Evolution. State University of New York.

Rohlf, F.J. 2008. *tpsRelw, relative warps analysis*, Version 1.46. Stony Brook NY: Department of Ecology and Evolution. State University of New York.

Rohlf, F.J. 2009. *tpsDig, digitize landmarks and outlines*, Version 2.14. Stony Brook NY: Department of Ecology and Evolution. State University of New York.

Wolak, M.E., D.J. Fairbairne and Y.R. Paulsen. 2012. Guidelines for estimating repeatability. Methods in Ecology Evolution 3:129-137.

Zelditch, M. L., D.L. Swiderski, D. M. Sheets and W.L. Fink. 2004. *Geometric Morphometrics for Biologists: A Primer*. Elsevier Academic Press, Oxford, UK.
